# Successful treatment of a blunt retrohepatic vena cava injury using an atriocaval shunt without sternotomy or thoracotomy: a case report

**DOI:** 10.3389/fsurg.2023.1152666

**Published:** 2023-05-05

**Authors:** Donghwan Choi, Dongmin Seo, Hang Jun Choi, Jonghwan Moon

**Affiliations:** Division of Trauma Surgery, Department of Surgery, Ajou University School of Medicine, Suwon, Republic of Korea

**Keywords:** blunt liver injuries, retrohepatic vena cava injury (RHIVC), total hepatic vascular isolation, atriocaval shunt, transdiaphragmatic incision

## Abstract

Retrohepatic vena cava (RHIVC) injury is often fatal and can be very difficult to manage. Total hepatic vascular isolation, a shunt, or bypass surgery is required for the surgical treatment of RHIVC injury in hemodynamically unstable patients; however, these are not easy procedures. Here, we present a case of RHIVC injury that was successfully treated by atriocaval shunt placement via a transdiaphragmatic incision without sternotomy or thoracotomy. In addition, we review the resuscitation and surgical procedures used for total hepatic vascular isolation in patients with RHIVC injury.

## 1. Introduction

Blunt liver injuries have a high mortality rate and are often associated with multiple injuries (1). Among these, injuries to the retrohepatic vena cava (RHIVC) are rare but fatal in most cases (2, 3). Access to the retrohepatic area in hemodynamically unstable patients with RHIVC injury is challenging and involves time-consuming procedures that cause significant bleeding (2). Several studies have reported the treatment of RHIVC injury using sternotomy and an infrarenal approach with a shunt (2, 3). Here we describe a case involving a 25-year-old man with RHIVC injury that was successfully treated with a time-saving approach involving atriocaval shunt placement via a transdiaphragmatic incision without median sternotomy or thoracotomy. In addition, we review procedures for complete hepatic isolation and exposure of the retroperitoneal area and suprahepatic vena cava for placement of a shunt in patients with RHIVC injury.

## 2. Case description

The patient was a 25-year-old man who experienced a fall and displayed signs of shock. Paramedics contacted the hotline of the trauma gatekeeper to arrange transport and provide medical guidance. The gatekeeper instructed the paramedics to secure intravenous (IV) access and administer sufficient crystalloids. The patient was transported from the accident scene to the trauma center in 20 min, and he presented with facial, scalp, and abdominal wall wounds.

### 2.1. Clinical findings

**Figure 1:**
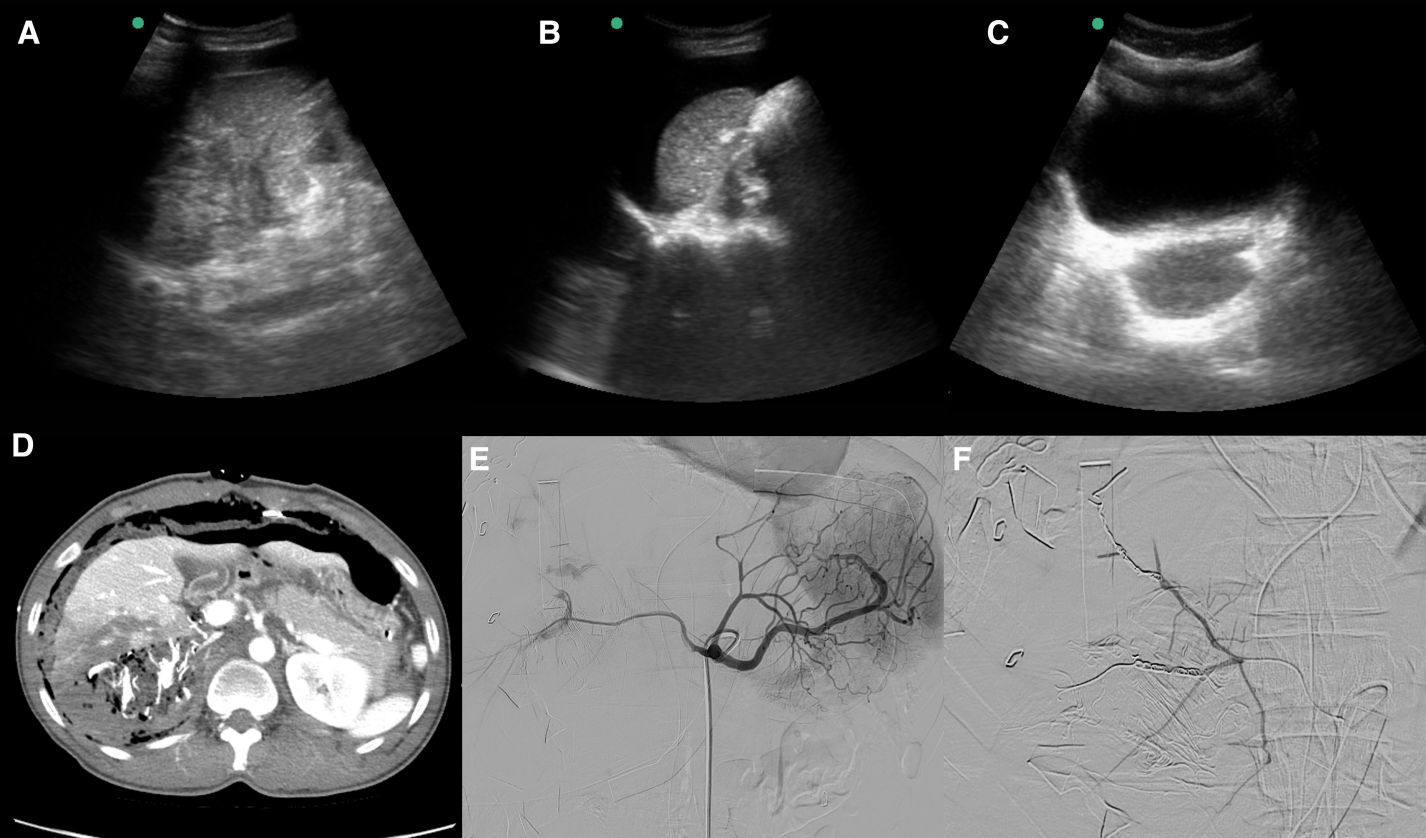
Focused assessment with sonography in trauma (FAST), postoperative computed tomography (CT), and angioembolization in a patient with a blunt retrohepatic vena cava injury. (**A**) FAST (Morrison's pouch, no free fluid). (**B**) FAST (perisplenic free fluid). (**C**) FAST (pelvic cavity, no free fluid). (**D**) Postoperative abdominal contrast-enhanced CT. (**E,F**) Postoperative angioembolization.

The initial vital signs were as follows: systolic/diastolic blood pressure, 74/57 mmHg; heart rate, 118/min; respiratory rate, 30/min; temperature, 34.6°C; SpO_2_, 99%; and Glasgow Coma Scale score, 13. Initial assessment was performed according to advanced trauma life support protocols, and crystalloid infusion, intubation, and massive transfusion were initiated. Focused assessment with sonography in trauma revealed a large amount of free fluid in the perisplenic area (Figure 1). Blood gas analysis revealed the following: pH, 7.186; lactic acid, 10.96 mmol/L; and base excess, −11.5 mmol/L. Laboratory findings revealed the following: hemoglobin, 12.7 g/dl; platelet, 205 × 10^3^/µl; prothrombin time, 18.3 s; international normalized ratio, 1.6; activated partial thromboplastin clotting time, 51 s; fibrinogen, 123 mg/dl; fibrin degradation products, 115 µg/ml; and D-dimer, 7.6 µg/ml.

### 2.2. Timeline

**Figure 2:**
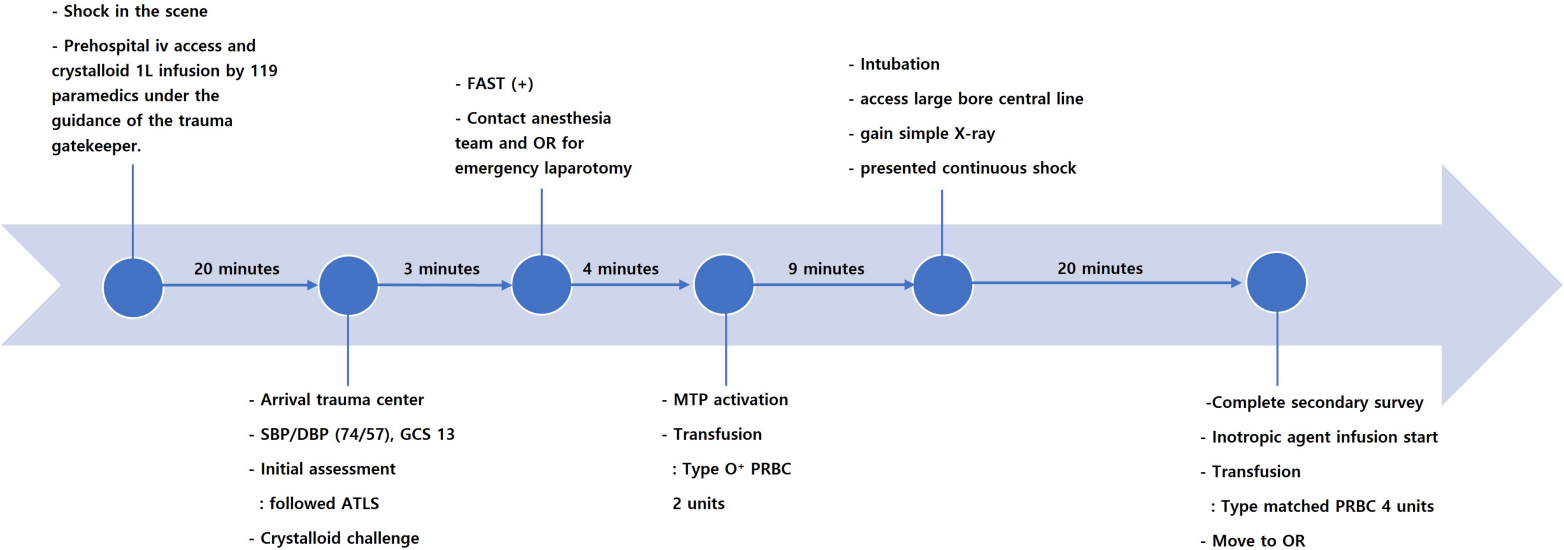
Flow of resuscitation from the accident site to the operating room for a patient with a blunt retrohepatic vena cava injury.

The chronological resuscitation process is shown in Figure 2. The patient was taken to the operating room within 38 min of arrival at the trauma center.

### 2.3. Therapeutic interventions

**Figure 3:**
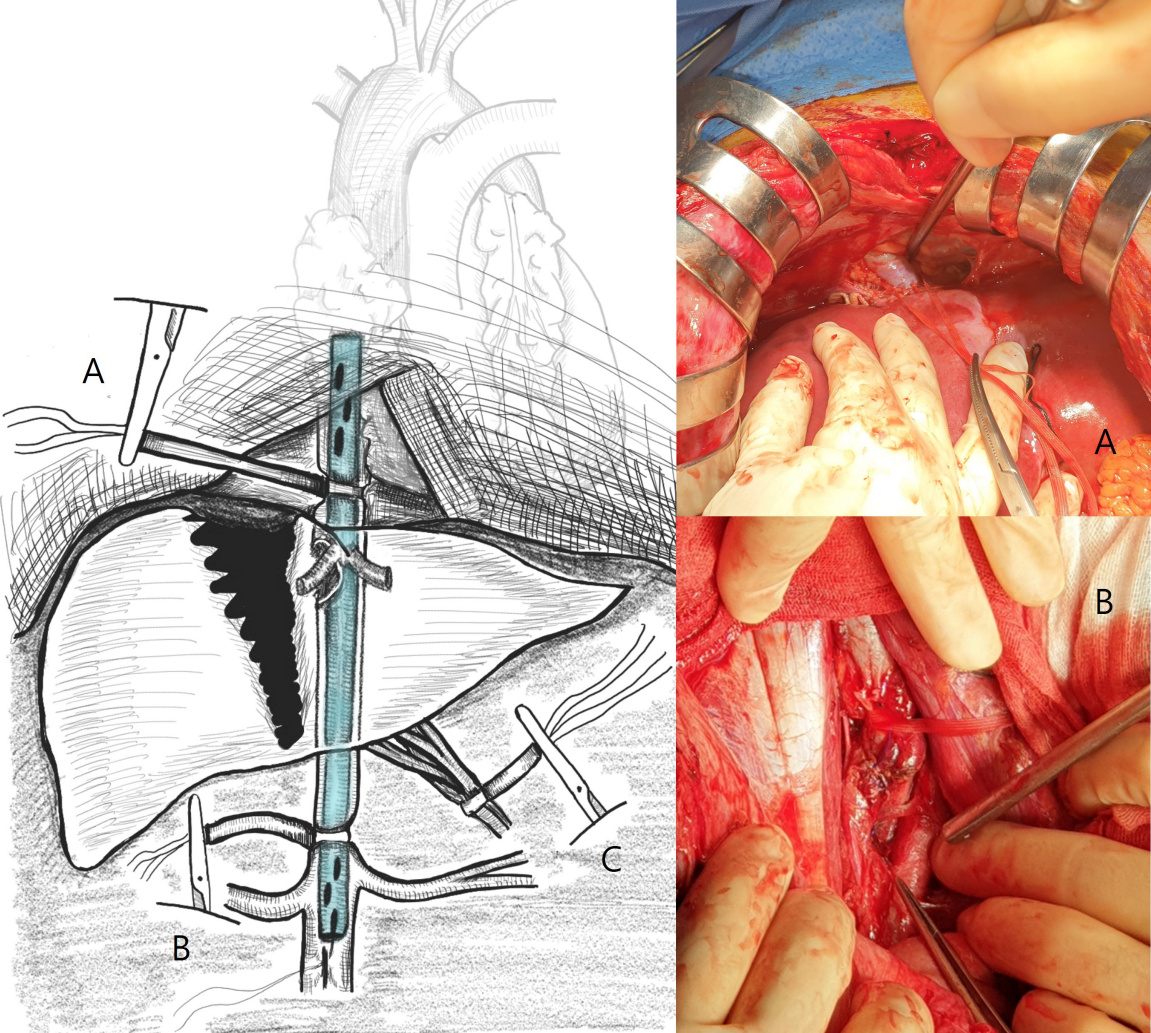
Total hepatic vascular isolation and atriocaval shunt placement via a transdiaphragmatic incision in a patient with a blunt retrohepatic vena cava injury. (**A**) Transdiaphragmatic incision and intrapericardial vena cava snaring. (**B**) Suprarenal vena cava snaring. (**C**) Pringle maneuver.

A long midline laparotomy incision was made, and massive hemoperitoneum and major hepatic lacerations were identified. The porta hepatis was clamped using the Pringle maneuver; however, the bleeding continued. The laparotomy incision was extended into the right subcostal incision and the perihepatic area was packed. Medial visceral rotation was performed to allow retroperitoneal exposure. A right renal hilum injury was identified, and total nephrectomy was performed. The infrarenal vena cava was injured and underwent primary repair. The suprarenal vena cava was dissected to prepare it for distal snaring. The infrapericardial region was incised through the abdominal cavity to create a pericardial window, and the intrapericardial inferior vena cava was exposed. After dissection around the intrapericardial inferior vena cava, proximal snaring was performed using a vascular loop. The upper part of the suprarenal vein was dissected to prepare it for snaring using a vascular loop. A 28-Fr chest tube was prepared for an atriocaval shunt, and the length from the right atrium (RA) to the suprarenal vena cava was measured. Following placement of a purse-string suture on the suprarenal vena cava, venotomy was performed. The chest tube was inserted into the inferior vena cava (IVC) to facilitate insertion of the suprarenal vena cava into the RA shunt, and the proximal and distal vena cava were snared. Simultaneously, total hepatic vascular isolation and liver mobilization were performed by clamping the porta hepatis using the Pringle maneuver (Figure 3). Rupture of the hepatic vein and RHIVC were confirmed, and primary repair with 4–0 prolene sutures was completed. The purse-string suture applied to the suprarenal IVC was released, and the chest tube was removed from IVC. Extensive liver parenchymal injury was identified and repaired with sutures. The durations of the Pringle maneuver were 17 and 13 min, respectively. Perihepatic packing, retroperitoneal packing, and temporary abdominal wall closure were performed, and the primary surgery was completed. During surgery, 24 units of packed red blood cells, 28 units of fresh frozen plasma, and 1 unit of platelet pheresis were transfused.

Angiography was performed immediately after the first surgery, and it revealed multiple contrast-enhanced extravasations from the right hepatic artery and right renal capsular artery. Subsequently, embolization was performed using a coil and glue (Figure 1).

After the first surgery, the patient's hemodynamic status improved. However, considering the drainage of 565 cc of additional blood, an increase in norepinephrine requirements to maintain a stable hemodynamic status, and a lactic acid level of 5.41 mmol/L, an additional bleeding site was suspected. Therefore, a second surgery was quickly scheduled and performed 16 h after the first one. Bleeding from RHIVC and the infrarenal vena cava that was observed during the first surgery was also observed during the second surgery, and additional sutures were placed under the Pringle maneuver. A primary liver suture was also added. Perihepatic packing, retroperitoneal packing, and temporary abdominal wall closure were performed, and the secondary surgery was completed. An atriocaval shunt was not placed.

The third surgery was performed 28 h after the second one, and there was no additional bleeding. The pericardial incision in the shunt was repaired. Abdominal wall closure was performed, and the third surgery was completed.

### 2.4. Follow-up and outcomes

The patient was diagnosed with ventilator-associated pneumonia and sepsis, supported by a ventilator, and administered antibiotics. Deep vein thrombosis and pulmonary thromboembolism were confirmed, and a non-vitamin K antagonist oral anticoagulant was administered. Surgical site infection was diagnosed, and surgical debridement and negative-pressure wound therapy were performed. Open reduction and internal fixation were performed for the facial bone, elbow, and femur fractures. The traumatic subdural hemorrhage resolved after conservative treatment. The patient was treated in the intensive care unit for 15 days and remained in the hospital for 44 days. The revised trauma score and injury severity score were 6.0848 and 59, respectively. At the time of writing this report, the patient was being followed up on an outpatient basis.

## 3. Discussion

RHIVC injury is reportedly fatal, showing the lowest survival rate among IVC injuries (4). Unlike penetrating injuries such as gunshot injuries, blunt RHIVC injuries often require surgery in the absence of information regarding the level of injury. This is because patients with IVC injury are often hemodynamically unstable and undergo surgery without imaging studies (5). Concomitant injuries are common in patients with RHIVC, and most patients exhibit high-grade liver injury (3). Contained RHIVC injury has been reportedly treated with nonoperative management or arterial stent grafting, although hemodynamically unstable patients require surgical treatment (6, 7). Surgeries for treating RHIVC injuries are very difficult. Venous return through IVC accounts for more than 75% of the cardiac output (8), and maintenance of an adequate systemic circulating blood volume is difficult when the damaged area is exposed for IVC repair and the distal IVC is blocked to secure an operating space. Moreover, exposure of the RHIVC injury area to surgical intervention in hemodynamically unstable patients can cause significant bleeding from the IVC and liver (2, 3). Therefore, for surgery in these patients, preservation of the blood volume returned to the RA through IVC using a shunt or bypass is necessary (3). Several studies have reported the treatment of RHIVC injury using sternotomy or thoracotomy and an infrarenal approach with a shunt or cardiopulmonary bypass (3, 9, 10). To perform shunting or bypass surgery, exposure of the retroperitoneal area using medial visceral rotation and heart exposure using sternotomy or thoracotomy are required. However, these techniques are difficult and time-consuming (2). Shunting or bypass surgery for repair require a large amount of resources and equipment and extensive communication with the anesthesia team. Repeated exposure to the bleeding focus for identification of a blunt RHIVC injury may lead to deterioration of the patient's physiological parameters, with disastrous outcomes (11). Therefore, the surgical team should perform the Pringle maneuver at an early stage in order to confirm RHIVC injury. If an RHIVC injury that is difficult to control with packing is confirmed, shunt or bypass-capable resources should be mobilized, and information about the surgical procedure should be delivered to the anesthesia and scrub teams. To prevent hemodynamic collapse during IVC repair, large-bore IV access is essential, with access above the level of the diaphragm preferred over femoral access (11). For total hepatic vascular isolation, stepwise implementation of the Pringle maneuver and infrahepatic and suprahepatic IVC control are required (2, 3). During the repair process, there is a high risk of air embolism through the shunt or a large defect in the IVC wall; therefore, caution is required during the procedure, and the anesthesia team should be aware of the possibility of its occurrence (11). Close follow-up is necessary after RHIVC repair because it can lead to IVC stricture or thromboembolism. To prevent delayed IVC strictures, a repair procedure using autologous veins, artificial grafts, or biological patches can be performed. However, these surgeries require a considerable amount of time; therefore, the decision should be made on the basis of the patient's hemodynamic reserve (11). In our case, without median sternotomy or thoracotomy, a pericardial window was created through a transdiaphragmatic incision, and an atriocaval shunt was created by snaring the intrapericardial IVC. We exposed the entire diaphragm and liver through a long midline incision with a subcostal extension. The length of the tube inserted into the shunt was determined by direct measurement of the proximal and distal snaring lengths. The surgical method involving a transdiaphragmatic incision was reportedly used for children with RHIVC injuries by Evans et al. (12). However, because RHIVC injury is rare, it is difficult to generalize the procedure described in this report. Another limitation is that access to the RA without thoracotomy or sternotomy may be problematic in obese or overweight patients. Therefore, this technique can be considered when a sufficient field of view for a transdiaphragmatic incision can be secured.

In summary, blunt RHIVC injuries are very rare and exhibit a high fatality rate, and challenging surgical procedures for damage control are required in such cases. For the successful treatment of blunt RHIVC injuries, timely detection and surgery using multidisciplinary resources, including trauma surgeons, cardiovascular specialists, anesthesia teams, scrubs, and radiology interventionists, are required. We believe that RHIVC injuries can be successfully treated using techniques that can facilitate surgery while maintaining the patient's hemodynamics, such as an atriocaval shunt, cardiopulmonary bypass, and complete hepatic vascular isolation.

## Data Availability

The original contributions presented in the study are included in the article, further inquiries can be directed to the corresponding author.
